# Exploring the psychological determinants of smoking behavior among immigrant university students in Italy: A convergent mixed-methods cross-sectional study

**DOI:** 10.18332/tid/220358

**Published:** 2026-05-28

**Authors:** Maryam Zarifkar, Antonio Francesco Maturo, Fatemeh Kochackpour

**Affiliations:** 1 Department of Quality of Life Studies, University of Bologna, Rimini, Italy; 2 Department of Health and Exercise Science, University of Oklahoma, Norman Campus, Norman, Oklahoma, United States

**Keywords:** immigrant students, smoking prevalence, mental health, mental toughness

## Abstract

**INTRODUCTION:**

Although smoking is not an effective coping mechanism, it is a common behavior among university students experiencing psychological distress, but limited research has examined its psychological determinants in multicultural university settings. This mixed-methods study investigated the relationship between smoking behavior and the mental toughness scale.

**METHODS:**

A convergent mixed-methods cross-sectional study design was conducted between March and June 2025 among 280 immigrant students at the University of Bologna. Eligible participants were students aged 18–30 years, currently enrolled in a study program, able to communicate in English or Italian, and without chronic conditions affecting smoking behavior. Quantitative data assessed demographic characteristics, smoking patterns, and mental toughness, assessed using the Mental Toughness Questionnaire (MTQ-18), which evaluates control, commitment, challenge, self-confidence, and underlying psychological and migration-related stressors. Qualitative data were obtained through semi-structured interviews exploring emotional coping, cultural adaptation, and smoking motivations. Data were analyzed separately and integrated during interpretation.

**RESULTS:**

Among the 280 participants, 42.86% were smokers and 57.14% were nonsmokers. No statistically significant associations were found between smoking status and demographic variables, including gender (χ^2^=0.66, p=0.417), age (χ^2^=6.70, p=0.152), parental smoking (χ^2^=0.00, p=1.000), and sibling smoking (χ^2^=0.07, p=0.789). Most smokers reported low to moderate cigarette consumption (1–10 cigarettes per day). Across all mental toughness dimensions, smokers demonstrated lower mean scores compared to non-smokers. For instance, overall mental toughness scores were lower among smokers (males: 2.57; females: 2.31) compared to non-smokers (males: 3.84; females: 3.74). Qualitative findings highlighted key psychological stressors, including identity disruption, anxiety, academic pressure, and social isolation, which contributed to smoking behavior as a perceived means of emotional regulation.

**CONCLUSIONS:**

Smoking behavior among immigrant students is shaped primarily by psychological vulnerability and migration-related stress rather than demographic characteristics. Integrating mental health support, stress-management resources, and culturally responsive interventions into smoking prevention programs may be essential for reducing smoking in immigrant student populations.

## INTRODUCTION

Tobacco use remains a leading preventable cause of disease and death worldwide^1^. In 2019, about 8 million deaths and 200 million disability-adjusted life years were lost globally, making tobacco use the leading cause of death among adult men^2^. Findings show that lifetime smokers who are not daily smokers have an approximately 72% increased risk of death compared with never smokers, and their average life expectancy is approximately 5 years shorter^3^.

Recent evidence highlights the high prevalence of smoking among young adults aged 18–24 years, particularly university students^4^. University students, especially those studying abroad, represent a population in transition, often exposed to new social environments, cultural norms, and stressors that may encourage experimentation with tobacco products and affect their coping mechanisms and emotional regulation^5^. Students may smoke in response to stressful events or emotional discomfort, often perceiving it as a form of temporary relief rather than an effective coping strategy^6^. Furthermore, according to an earlier study, approximately 31.9% of undergraduate smokers reported that they smoke to help manage symptoms of depression^7^.

Research indicates that tobacco withdrawal is strongly associated with psychomotor restlessness, agitation, and heightened anxiety, which can intensify smoking urges among young adults^8^. Therefore, understanding the relationship between psychological factors and smoking behavior, and how students from diverse national backgrounds manage stress, social belonging, and personal control, is crucial for addressing tobacco use in higher education settings.

A growing body of research highlights the role of mental toughness, psychological resilience, emotional regulation, and self-control in shaping health-related behaviors among young adults^9,10^. These constructs, which are closely related to psychological resilience and mental toughness, reflect an individual’s ability to cope with adversity, maintain goal-directed behavior, and recover from setbacks^11^. Studies have linked stress and low self-esteem to teenage smoking, finding that children with low self-esteem are more likely to smoke than children with high self-esteem^12^. In addition, depression has been known to be a predictor of smoking initiation, particularly among adolescents who may become dependent on nicotine^13^. However, few studies have examined how these psychological characteristics intersect with smoking behaviors among multicultural university populations^14,15^. Existing evidence remains limited to single-ethnic contexts, leaving an important gap in understanding cross-cultural patterns of psychological determinants of smoking among immigrant students^16^.

Given these considerations, the present study employs a mixed design to investigate the psychological determinants of smoking behavior among university students from diverse national backgrounds. The quantitative section examines the relationship between smoking status and key psychological variables such as control, commitment, challenge, self-confidence, and mental toughness. The qualitative phase complements these findings by examining students’ lived experiences and coping narratives in stress management and social adjustment. This integrated approach aims to build a detailed understanding of smoking behavior in culturally diverse university settings and provide implications for community-based preventive interventions.

## METHODS

### Study design

This study used a convergent mixed-methods crosssectional study that integrated quantitative and qualitative approaches to provide a comprehensive understanding of the psychological determinants of smoking behavior among 280 immigrant students at the University of Bologna between March and June 2025.

### Participants and setting

This study was conducted among international and immigrant students at the University of Bologna. The sample size was determined based on feasibility considerations, including participant accessibility and the time frame of the study. No formal *a priori* power calculation was conducted. Participants were recruited through university announcements and online communication channels targeting international and immigrant students, including Telegram groups. Interested students volunteered to participate and were screened according to the predefined eligibility criteria: current enrollment at the University of Bologna, age 18–30 years, and ability to communicate in English or Italian. Smoking status was assessed by self-report, and respondents were categorized as current smokers or non-smokers. The final sample size of 280 participants, including 120 smokers and 160 non-smokers, was considered adequate to explore group differences in smoking behavior and psychological variables. Students with chronic physical or mental illnesses that affected smoking behavior were excluded.

The sample represented a culturally diverse group, including participants from the Middle East, North Africa, South Asia, Eastern Europe, and other regions. However, as participants were recruited through convenience sampling and voluntary participation, no specific strategy was implemented to ensure proportional representation of the broader immigrant student population at the University of Bologna. This study was conducted in accordance with the ethical principles of the Declaration of Helsinki. Participation was voluntary and informed written consent was obtained from all participants prior to data collection. All data were collected anonymously, and confidentiality was strictly maintained.

According to institutional guidelines, formal ethical approval was not required for this type of minimalrisk study involving voluntary participation and anonymous data collection among adult students. The study protocol, survey instruments and interview guides were reviewed and approved by the Department of Quality of Life Studies Group at the University of Bologna.

### Measures

The quantitative questionnaire consisted of three main sections; the first section assessed sociodemographic and behavioral information, including age, gender, nationality, smoking status (current vs non-smoker), and family history of smoking (parents/siblings). The second section assessed smoking-related behaviors, including smoking frequency. Daily cigarette consumption was categorized into four groups based on the number of cigarettes smoked per day: 1–5, 5–10, 10–15, and 15–20 cigarettes. For interpretative purposes, consumption levels were described as low to moderate when participants reported smoking between 1 and 10 cigarettes per day. These categories were defined based on the distribution of responses within the study sample and were not based on predefined clinical thresholds.

The third section assessed psychological determinants such as control, commitment, challenge, and self-confidence, measured using the standard mental toughness instrument. The Mental Toughness Questionnaire (MTQ18) is an 18-item scale. It consists of four subscales that focus on personal, social, and academic self-esteem^17^. The reliability coefficient of this questionnaire, established in 2005, showed Cronbach’s alpha ranging from 0.70 to 0.91^18^. The full questionnaire is provided in the Supplementary file.

For the qualitative component, a purposive sampling strategy was initially used to ensure diversity in gender, nationality, and smoking status. Participants were recruited through the same channels as the quantitative phase, and additional participants were identified through peer referral (snowball sampling), which facilitated access to a broader range of experiences within the immigrant student community^19^.

A total of 30 participants were included, as this number was considered sufficient to achieve data saturation, defined as the point at which no new themes or meaningful insights emerged from additional interviews. The use of snowball sampling further supported the inclusion of participants with varied backgrounds and experiences, enhancing the depth and richness of the qualitative data. Interview questions explored perceived motivations for smoking or quitting, emotional coping, cultural adaptation, and self-control in stressful situations. Sessions were held in person or online in student settings. Each session lasted approximately 30–45 minutes. Interviews were recorded and transcribed verbatim with the participants’ consent.

### Data analysis

Quantitative data were analyzed using SPSS version 29. Descriptive statistics, frequencies, and percentages were calculated for all sociodemographic and behavioral variables^20^. The association between smoking status and categorical variables such as gender and family history of smoking (parents/ siblings) was examined using chi-squared tests.

Independent t-tests and one-way ANOVA were performed to compare total scores and subscale scores of the Mental Toughness Questionnaire (MTQ18) for control, commitment, challenge, and confidence, across smoking groups. All statistical tests were two-tailed, and a significance level of p<0.05 was considered.

For qualitative data analysis, transcribed interviews were read several times to ensure familiarity with the data, and initial codes were generated inductively. Codes were then organized into broader themes that represented key psychological and cultural dimensions associated with smoking behavior, such as stress regulation, coping strategies, social belonging, and self-esteem and self-control, and were analyzed using MAXQDA 24 software^21^. Data from the quantitative and qualitative sections were subsequently integrated through triangulation, allowing for the relationship between statistical findings on mental toughness and participants’ narrative experiences of coping, adaptation, and emotional regulation in multicultural student environments.

## RESULTS

Table 1 shows the distribution of smoking and nonsmoking students by key demographic characteristics. Among the 280 students, 120 (42.86%) were smokers, and 160 (57.14%) were non-smokers. Male students had a higher number of smokers (66) compared to female students (54). A similar pattern was observed among non-smokers, with 79 males and 81 females, indicating that the distribution of smoking behavior was relatively balanced across genders.

**Table 1:** Demographic characteristics of smoking and non-smoking immigrant university students, convergent mixed-methods cross-sectional study, University of Bologna, Italy, 2025 (N=280)

*Characteristics*	*Smokers (N=120)* *n (%)*	*Non-smokers (N=160)* *n (%)*
**Gender**		
Male	66 (27.1)	79 (28.2)
Female	54 (15.7)	81 (28.9)
**Age** (years)		
18–19	7 (5.8)	20 (12.5)
20–22	36 (30.0)	36 (22.5)
23–25	46 (38.3)	57 (35.6)
26–28	21 (17.5)	25 (15.6)
29–30	10 (8.3)	22 (13.7)
**Smoker parents**	14 (11.6)	18 (11.2)
**Non-smoker parents**	106 (88.3)	142 (88.7)
**Smoker siblings**	10 (8.3)	16 (10.0)
**Non-smoker siblings**	110 (91.6)	144 (90.0)

The highest proportion of smokers was found in the 23–25 years age group (46 students), followed by the 20–22 age group (36 students) and the lowest number of smokers was in the 18–19 age group (7 students).

Most smokers (106 out of 120) reported having non-smoking parents, while only 14 had smoking parents. A similar pattern was observed among nonsmokers, indicating that most students came from families where neither parent smoked. Most smokers (110 students) reported having non-smoking siblings, with only 10 indicating a smoking sibling. The same trend was observed among non-smokers as well.

Table 2 summarizes the results of the chi-squared test examining the relationship between smoking status and several demographic variables. This analysis did not reveal any statistically significant associations across all variables. The association between gender and smoking status was not substantial (χ^2^=0.66, df=1, p=0.417). Age groups also did not show a significant relationship with smoking status (χ^2^=6.70, df=4, p=0.152). Although some age groups had a higher frequency of smokers, these differences were not statistically significant. The presence of a parent who smoked was not associated with students’ smoking behavior (χ^2^=0.00, df=1, p=1.000). Smoking and non-smoking students were similarly distributed across parental smoking categories. Similarly, having a smoking sibling did not show a significant association with smoking status (χ^2^=0.07, df=1, p=0.789).

**Table 2:** Chi-squared test results for the association between demographic variables and smoking status among immigrant university students, convergent mixed-methods cross-sectional study, University of Bologna, Italy, 2025 (N=280)

*Variables*	*χ^2^*	*p*
Gender × Smoking	0.66	0.417
Age × Smoking	6.70	0.152
Parental × Smoking	0.00	1.000
Siblings × Smoking	0.07	0.789

Table 3 shows the distribution of daily cigarette consumption among male and female student smokers. Overall, most smokers reported low to moderate daily consumption. Among male smokers (n=76), the highest proportion reported daily consumption of 1–5 cigarettes (34.21%), followed by the same pattern among female smokers (n=44), with the highest percentage in the 1–5 cigarettes per day category (31.82%). The chi-squared test examining the association between gender and daily cigarette consumption categories indicated no statistically significant relationship (χ^2^=2.92, df=3, p=0.404).

**Table 3:** Distribution of daily cigarette consumption among smoking immigrant university students by gender, convergent mixed-methods cross-sectional study, University of Bologna, Italy, 2025 (N=280)

*Cigarettes per day*	*Male students* *(N=76)* *n (%)*	*Female students* *(N=44)* *n (%)*
1–5	26 (34.2)	14 (31.8)
5–10	21 (27.6)	8 (18.1)
10–15	12 (15.7)	12 (27.2)
15–20	17 (22.3)	10 (22.7)

Table 4 presents the average mental toughness scores among smoking and non-smoking students across the four MTQ18 components. In all subscales, control, commitment, challenge, and confidence, nonsmokers demonstrated higher mean scores compared to smokers. Among smokers, males showed slightly higher mental toughness than females (2.57 vs 2.31). In contrast, non-smoking males exhibited the highest overall mental toughness (3.84), followed by nonsmoking females (3.74). These patterns may suggest a potential relationship; however, further statistical analysis is required to confirm this association.

**Table 4:** Mean mental toughness scores (MTQ-18) among smoking and non-smoking immigrant university students, convergent mixed-methods cross-sectional study, University of Bologna, Italy, 2025 (N=280)

*Component*	*Male smokers* *Mean ± SD*	*Female smokers* *Mean ± SD*	*Male non-smokers* *Mean ± SD*	*Female non-smokers* *Mean ± SD*
Control	2.45 ± 0.54	2.33 ± 0.49	3.60 ± 0.35	3.88 ± 0.36
Commitment	2.56 ± 0.25	2.54 ± 0.20	3.75 ± 0.30	3.74 ± 0.36
Challenge	2.61 ± 0.41	2.06 ± 0.64	3.88 ± 0.13	3.68 ± 0.31
Confidence	2.66 ±0.42	2.31 ± 0.53	4.15 ± 0.23	3.65 ± 0.38
Mental toughness	2.57 ± 0.08	2.31 ± 0.05	3.84 ± 0.32	3.74 ± 0.40

### Qualitative results: psychological factors

*Identity and emotional conflict*

The migration process often created a conflict between maintaining native cultural values and adapting to new social expectations in Italy. Many students reported a profound sense of identity confusion and emotional conflict. These experiences frequently undermined their self-esteem and diminished their perceived sense of control, two elements that closely align with the lower scores observed in the ‘control’ and ‘confidence’ dimensions of the mental toughness scale among smokers.

Students described smoking not only as a strategy for managing emotional discomfort but also as a symbolic behavior that helped them navigate this identity tension. For some, smoking represented rebellion, personal freedom, or a way to project confidence in unfamiliar social contexts. These perceptions mirror the lower quantitative scores in the ‘challenge’ and ‘commitment’ components, suggesting that emotional and cultural conflict may weaken psychological resilience and increase reliance on smoking as a coping mechanism. A participant said:

*‘At home, I never smoked in public. My parents never allowed me. But here, I can do whatever I want, and maybe I’m doing it too much now, just because no one is watching.’*

Several students also noted that Italian youth culture, with its more permissive views on smoking and personal independence, influenced their behavior and reinforced this newfound autonomy. Participant (SS 7) stated:

*‘Italy was a new home for me, where everything felt free. This duality scared me at first because I no longer had a sense of external control. Later, I let myself go and smoked freely.’*

This exposure to a more liberal social environment appeared to validate their choices, even when these behaviors conflicted with the values they had grown up with. The emotional and cultural tension between freedom and guilt, and between identity exploration and conformity, often deepened psychological dependence on smoking not only as a physical habit but as a symbol of belonging, maturity, and personal independence. Combined with feelings of isolation and cultural displacement, this conflict contributed to a pattern consistent with the lower mental toughness scores observed quantitatively among smokers, particularly in control, confidence, and challenge.

*Anxiety and mental health concerns*

Many participants reported struggling with significant anxiety and a range of underlying emotional challenges, including symptoms of depression, separation anxiety, generalized anxiety, academic performance anxiety, social anxiety, and both pre- and post-migration stress. These psychological burdens closely align with the lower scores observed in the ‘challenge’ and ‘confidence’ components of the mental toughness scale among smokers in the quantitative phase. Several students noted that they had experienced mild to severe anxiety before arriving in Italy, but had never had the opportunity or resources to address these concerns before migration.

The migration process itself intensified these difficulties. Students described the transition to Italy as emotionally overwhelming, noting cultural adaptation, language barriers, bureaucratic uncertainties, social isolation, and pressure to perform academically within a new educational system. These conditions often exacerbated pre-existing anxiety and diminished their perceived ability to cope with daily challenges, mirroring the reduced psychological resilience reflected in the quantitative findings.

Many students expressed a sense of disconnection from their usual support systems, particularly family, and were unsure how to access mental health services within the Italian healthcare system. In this context, smoking emerged as a primary coping strategy used to regulate anxiety, depressive symptoms, or emotional distress. Participant (SS 10) said:

*‘When things get out of hand, a few minutes of smoking gives me a sense of calm. It’s the only time I feel like I can breathe.’*

Beyond nicotine’s physiological effects, participants emphasized the psychological meaning of the smoking ritual, stepping outside, taking a break, being alone, or distancing themselves from academic and social pressures. These moments were often described as a way to regain a sense of control when external demands felt unpredictable or overwhelming. This experience parallels the lower ‘control’ scores observed quantitatively among smokers.

For many, smoking functioned as an emotional anchor, a temporary escape from overthinking, homesickness, or psychological strain. In this sense, smoking became not only a behavioral habit but a self-directed mental health tool, despite its longterm consequences. These patterns reinforce the quantitative finding that smoking may be used as a compensatory mechanism in the context of weakened psychological resilience, particularly reduced confidence and challenge-orientation among smokers.

These insights highlight the importance of integrating mental health support into smoking prevention and cessation programs for immigrant populations in Italy, who may face heightened vulnerability to emotional distress and limited access to culturally responsive psychological services.

*Stress and emotional coping*

Survey and interview findings consistently indicated that students viewed smoking as a primary coping mechanism for managing stress and emotional overload. This interpretation is supported by the quantitative data showing that most smokers consumed cigarettes in the lower daily range (1–10 cigarettes), suggesting that smoking functioned largely as a stress-relief behavior rather than dependence-driven heavy use. The preference for light to moderate consumption aligns with a pattern of situational coping, particularly during periods of acute psychological strain.

Many students at the University of Bologna described experiencing moderate to high levels of psychological stress because of migration-related challenges, including separation from family support networks, the need to rebuild daily life from the ground up, navigating unfamiliar administrative and social systems in Italy, and adapting to academic pressures in a foreign language. These stressors closely reflect the lower scores on the ‘control’ and ‘challenge’ components of the mental toughness scale among smokers, suggesting weakened coping capacity and reduced resilience in stressful environments.

Students repeatedly described smoking as the most immediate and accessible strategy during moments of emotional tension. Lighting a cigarette became an automatic response to uncertainty, academic pressure, interpersonal stress, or moments of isolation, a behavior that aligns with the lower ‘confidence’ scores observed in the quantitative findings. A participant stated:

*‘When I feel nervous about exams or being alone, I go outside to smoke. It clears my mind, even if it’s just for a few minutes, and I must admit I have no intention of quitting.’*

and Participant (SS 25) said:

*‘Smoking calms me down; I always smoke more when I’m nervous or upset. I once finished a pack of cigarettes in one night.’*

another participant also said:

*‘I was very stressed early in my immigration, especially when I was looking for a place to live. I was alone, I had no friends, I felt very sad, and all I could do was smoke.’*

Several participants also believed that nicotine helped them remain calm or focused during difficult moments. For these individuals, smoking was not perceived as a harmful habit but rather as a functional tool for emotional regulation. This perception, combined with reduced confidence and a diminished sense of control, corresponds with the quantitative pattern of lower mental toughness among smokers and helps explain the limited motivation to quit despite awareness of long-term health risks.

Overall, these results indicate a strong link between emotional coping processes and smoking among immigrant students in Italy. The results emphasize the importance of including stress management and mental health support programs in smoking prevention and cessation programs for immigrant populations, especially those who may be more at risk due to cultural displacement, lack of support networks, and lower levels of psychological well-being.

*Smoking cessation attempts and commitment*

Many participants reported attempting to reduce or quit smoking at various points during their time in Italy, yet most described difficulty maintaining these efforts over time. Students often expressed initial motivation to stop smoking, especially during periods of improved mental health or academic focus. Still, these attempts frequently collapsed when stress increased or when they encountered challenges related to migration and daily adjustment. This pattern mirrors the lower scores observed on the ‘commitment’ subscale of the mental toughness scale among smokers, indicating reduced persistence and difficulty sustaining long-term behavioral goals.

Participants described several barriers that prevented them from maintaining cessation efforts, including academic pressure, loneliness, friends who smoked, emotional fatigue, and uncertainty surrounding their future in Italy. For many, smoking was perceived as an immediate, reliable source of relief during stressful moments, undermining sustained commitment to quitting. Participant (SS 14) said:

*‘I tried to quit smoking many times, but every time something stressful happened, exams, problems at home, or feeling lonely, I started again immediately.’*

A participant stated:

*‘When life becomes overwhelming, quitting is the last thing on my mind. I don’t have the energy to fight with myself.’*

The absence of a stable support system in Italy further contributed to these failed attempts. Without consistent emotional support or structured guidance, students struggled to maintain the self-discipline required for cessation. For some, quitting felt impossible because smoking had become deeply embedded in their coping routines.

These qualitative findings are also consistent with the quantitative findings regarding lower commitment, and they suggest that the difficulty with sustaining behavioral goals may be an important psychological factor in the ongoing smoking behavior of immigrant students. Again, the implications are for the development of interventions that address not only nicotine dependence but also the emotional, social, and structural obstacles to commitment to quitting.

## DISCUSSION

The findings of this convergent mixed-methods cross-sectional study suggest important insights into the psychological and contextual factors associated with smoking behavior among immigrant students in Italy. Quantitative results showed that no statistically significant associations were observed between demographic variables such as gender, age, parental smoking, and sibling smoking and smoking status. However, these outcomes should be interpreted with caution, as the analysis was based on bivariate comparisons and did not account for potential confounding considerations. Therefore, the lack of significant associations does not necessarily imply a lack of demographic impact.

In contrast, the qualitative results emphasized a multiplex set of psychological, cultural, and migrationrelated factors that may provide additional insight into why smoking emerges and continues among this group.

Psychological factors, including emotional distress, anxiety, and stress, play a central role in shaping smoking behaviors among immigrant students^22,23^. The migration process often exposes individuals to substantial psychological pressures, such as cultural adjustment, academic demands, homesickness, and social isolation^24,25^.

A major theme emerging from the interviews was the role of identity disruption and emotional conflict during the migration process. Students repeatedly described conflicts between maintaining their original cultural values and adapting to new social norms in Italy. This tension often challenged their sense of control and self-confidence patterns that are closely associated with the lower scores on the ‘control’ and ‘confidence components of the mental toughness scale among smokers. These outcomes are consistent with previous research indicating that migrationrelated identity challenges can weaken psychological resilience and increase susceptibility to maladaptive coping behaviors^26,27^.

Anxiety and mental health concerns also played a central role^28,29^. Many students reported experiencing various forms of anxiety, such as separation anxiety, generalized anxiety, academic pressure, and social anxiety. These emotional challenges were often increased by the stressors of adapting to life in Italy, such as navigating unfamiliar structures, facing language barriers, and feeling disconnected from familiar support networks. These experiences match the lower ‘challenge’ scores observed quantitatively among smokers, suggesting that anxiety reduces their perceived capacity to manage difficult situations. The association between qualitative narratives and quantitative mental toughness measures supports the interpretation that psychological vulnerability may be a key driver of smoking behavior in this population.

The integrative answers further demonstrated that smoking served primarily as a stress-management strategy rather than a pattern of heavy nicotine dependence. Quantitative data showed that most smokers fell within the low-consumption range (1– 10 cigarettes per day). At the same time, qualitative accounts showed that smoking was used to regulate emotional distress, restore a momentary sense of control, and provide psychological relief during periods of stress or uncertainty. The ritualistic aspects of smoking, stepping outside, being alone, or briefly disengaging from stress, were repeatedly emphasized by participants as meaningful components of their emotional regulation. These insights support existing literature suggesting that smoking can function as a coping behavior among individuals with limited access to mental health resources^30,31^.

The qualitative accounts also helped explain the lower scores on the ‘commitment’ subscale observed among smokers in the quantitative phase. Several students described repeated but unsuccessful attempts to quit smoking, noting that their efforts quickly collapsed when faced with academic stress, emotional strain, or feelings of isolation. These gaps suggest difficulty sustaining long-term behavioral goals, a pattern consistent with reduced commitment. The lack of stable support networks in Italy and the reliance on smoking as an immediate coping tool further weakened persistence, reinforcing the interpretation that limited psychological resilience contributes to ongoing smoking behavior among immigrant students^32,33^. Also, greater life satisfaction directly reduces the chances of trying smoking again^34^. Satisfying support from family, friends, and local communities appears protective, enhancing resilience and reducing the possibility of starting or maintaining smoking^35,36^.

### Implications for policies and research

Importantly, it is worth noting that the Italian context also played a crucial role in shaping these behaviors. The students described how they were exposed to more permissive social attitudes towards smoking, more independence, and less external monitoring. Smoking may have been a symbol of independence, adulthood, or being part of Italian youth culture. The cultural exposure may have validated these smoking behaviors even when they went against the values that they had internalized before coming to Italy. The results indicate that cultural displacement and adaptation pressures may interact with emotional vulnerability to enhance smoking as identity and stress-reflective behaviors.

Overall, these results indicate that there is potential for smoking prevention and cessation strategies to go beyond traditional risk factor approaches and to take into consideration the psychological and cultural realities of immigrant youth. Smoking prevention and cessation strategies may benefit from exploring ways to incorporate mental health support, stress management skills, culturally sensitive counseling, and programs to help youth cope with the demands of acculturation and identity reconstruction. The psychological strain may be essential in helping to alleviate smoking as a coping mechanism, especially for immigrant youth without access to support networks during this transition to life in Italy.

### Limitations

This study has several limitations. First, the crosssectional design does not allow for causal inference, and therefore, the observed associations should not be interpreted as cause-and-effect relationships. Second, the sample was restricted to immigrant students from a single university, which may limit the generalizability of the findings.

In addition, the use of convenience sampling and voluntary participation may have introduced selection bias and limited the representativeness of the sample. Although efforts were made to include participants from diverse cultural backgrounds, no specific strategy was implemented to ensure proportional representation of the broader immigrant student population.

Furthermore, the quantitative analysis was based on bivariate statistical tests and did not include multivariable regression models. As a result, potential confounding factors were not controlled for, and residual confounding cannot be excluded. Therefore, the findings should be interpreted with caution.

Self-reported data on smoking behavior and psychological variables may also be subject to recall bias and social desirability bias. Additionally, the qualitative sample, although rich in detail, may not fully capture the diversity of experiences across different immigrant groups.

### Future research

Future research should include larger and more diverse samples across multiple university settings and employ longitudinal designs to better examine the temporal relationships between psychological resilience, acculturation stress, and smoking behavior. Moreover, studies incorporating multivariable analytical approaches and evaluating culturally tailored mental health and smoking cessation interventions are needed to strengthen the evidence base and inform more effective prevention strategies.

## CONCLUSIONS

This study indicates that the smoking behavior of immigrant students in Italy is shaped by psychological rather than demographic factors. The results of this study indicate that there is a significant relationship between mental toughness factors such as control, confidence, challenges, and commitment among immigrant students in Italy. However, the qualitative study indicates that there is an identity conflict among these students. These students might use smoking as a coping strategy because of cultural adjustment problems, isolation, academic demands, and a lack of mental health services in Italy. These factors might lead them to heavily rely on smoking as a coping strategy. These findings indicate that there is a need to provide mental health services in order to prevent smoking among immigrant students in Italy.

## Supplementary Material



## Data Availability

The data supporting this research are available from the authors on reasonable request.
