# Supplementary material for: Exploring the psychological determinants of smoking behavior among immigrant university students in Italy: A convergent mixed-methods cross-sectional study
Source: Tob Induc Dis. 2026 May 28;24:10.18332/tid/220358. doi: 10.18332/tid/220358 (PMC13218404; doi:10.18332/tid/220358)
Supplement: Supplementary file 1 [file TID-24-76-s1.pdf]

Mental toughness questionnaire (MTQ-18)

Instruction: Please rate how true each statement is for you using the scale below:
1 = Strongly Disagree 2 = Disagree 3 = Neutral 4 = Agree 5 = Strongly Agree

Item	1	2	3	4	5
1. I generally feel in control of my life.	☐	☐	☐	☐	☐
2. I can usually find a solution when faced with a difficult problem.	☐	☐	☐	☐	☐
3. I see challenges as opportunities for growth.	☐	☐	☐	☐	☐
4. I remain calm under pressure.	☐	☐	☐	☐	☐
5. I am confident in my ability to achieve my goals.	☐	☐	☐	☐	☐
6. I can cope well with unexpected changes.	☐	☐	☐	☐	☐
7. I bounce back quickly after setbacks.	☐	☐	☐	☐	☐
8. I consistently push myself to improve.	☐	☐	☐	☐	☐
9. I stay focused even when things get difficult.	☐	☐	☐	☐	☐
10. I do not give up easily.	☐	☐	☐	☐	☐
11. I believe I can influence what happens in my life.	☐	☐	☐	☐	☐
12. I handle criticism well.	☐	☐	☐	☐	☐
13. I stay motivated even when progress is slow.	☐	☐	☐	☐	☐
14. I maintain a positive attitude during tough times.	☐	☐	☐	☐	☐
15. I take responsibility for my actions.	☐	☐	☐	☐	☐
16. I keep going even when I don’t feel like it.	☐	☐	☐	☐	☐
17. I trust my ability to handle whatever comes my way.	☐	☐	☐	☐	☐
18. I am committed to my long-term goals.	☐	☐	☐	☐	☐

© 2026 Zarifkar M. et al.
